# Optimization of the Extraction of Anthocyanins from the Fruit Skin of *Rhodomyrtus tomentosa* (Ait.) Hassk and Identification of Anthocyanins in the Extract Using High-Performance Liquid Chromatography-Electrospray Ionization-Mass Spectrometry (HPLC-ESI-MS)

**DOI:** 10.3390/ijms13056292

**Published:** 2012-05-22

**Authors:** Guo-Ling Liu, Hong-Hui Guo, Yuan-Ming Sun

**Affiliations:** 1 Department of Food Science, Yingdong College of Bioengineering, Shaoguan University, Shaoguan 512005, China; E-Mail: zibeike81@163.com; 2 College of Food, South China Agricultural University, Guangzhou 51000, China; E-Mail: ymsun@scau.edu.cn

**Keywords:** anthocyanins, *Rhodomyrtus tomentosa* (Ait.) Hassk, response surface methodology, HPLC-ESI-MS

## Abstract

Anthocyanins are naturally occurring polyphenols that impart bright color to fruits, vegetables and plants. In this study, the extraction of anthocyanins from freeze-dried fruit skin of downy rose-myrtle (*Rhodomyrtus tomentosa* (Ait.) Hassk var. Gangren) was optimized using response surface methodology (RSM). Using 60% ethanol containing 0.1% (v/v) hydrochloric acid as extraction solvent, the optimal conditions for maximum yields of anthocyanin (4.358 ± 0.045 mg/g) were 15.7:1 (v/w) liquid to solid ratio, 64.38 °C with a 116.88 min extraction time. The results showed good fits with the proposed model for the anthocyanin extraction (*R*^2^ = 0.9944). Furthermore, the results of high-performance liquid chromatography-electrospray ionization-mass spectrometry (HPLC-ESI-MS) analysis of the anthocyanins extracted from the fruit skin of downy rose-myrtle revealed the presence of five anthocyanin components, which were tentatively identified as delphinidin-3-glucoside, cyanidin-3-glucoside, peonidin-3-glucoside, petunidin-3-glucoside and malvidin-3-glucoside.

## 1. Introduction

Anthocyanins are the principal water-soluble pigments responsible for the red, blue, and purple colors of terrestrial plants. Anthocyanins are omnipresent in our plant-based diet, have little or no known toxicity [1], making them particularly attractive as natural substitutes for synthetic pigments and antioxidants [2]. In addition, an increasing number of studies have demonstrated that anthocyanins have the ability to prevent chronic and degenerative diseases including type 2 diabetes, cardiovascular disease and cancer [3–5].

*Rhodomyrtus tomentosa* (Ait.) Hassk, commonly known as downy rose-myrtle, is a small shrubby tree with pink flowers, and mainly distributed in south-east Asian countries, especially southern parts of China, Japan and Thailand [6]. The juicy berry of downy rose-myrtle turns dark purple when ripe, and is historically regarded as a healthy food and used for making traditional wine, jams and beverages. In downy rose-myrtle fruit, pigments are mainly located in the skin section, which is characterized as dark purple to black in color and probably represents a mixture of anthocyanins [7,8]. However, little information is available concerning the chemical constituents and pharmacologic activity of the downy rose-myrtle. In particular, the anthocyanin compounds in the fruit skin have not been fully elucidated.

Response surface methodology (RSM) is an effective statistical method based on a multivariate non-linear model, and has been widely used for optimizing complex process variables [9]. Several studies on optimizing conditions for the extraction of anthocyanins from different plants using RSM have been published [10,11]. These studies showed that RSM is useful for developing, improving and optimizing processes. In this study, the extraction of anthocyanins from the skin of downy rose-myrtle variety “Gangren” was optimized by applying RSM of three-variable, three-level Box-Behnken design (BBD), aiming to evaluate the influence of factors such as temperature, liquid-solid ratio, and time on the process of extraction. In addition, anthocyanins in the fruit skin of downy rose-myrtle were identified by high-performance liquid chromatography-electrospray ionization-mass spectrometry (HPLC-ESI-MS). The crude extract obtained can be used as food colorants, parapharmaceutical products or for further isolation and purification of a specific anthocyanin. The results of this study will provide valuable information for the exploitation of downy rose-myrtle resources.

## 2. Results and Discussion

### 2.1. Optimization of the Anthocyanin Extraction

Table 1 presents a Box-Behnken design with 17 experiments as well as the experimental and predicted response function. The predicted second-order polynomial model for the anthocyanin yield (Y) fitted in terms of actual factors (X_1_, temperature; X_2_, liquid-solid ratio; X, time) is shown as

(1) $$\begin{array}{l} Y=-44.72094+1.55138X_{1}+1.83475X_{2}+0.40796X_{3}+0.02155X_{1}X_{2}-0.003625X_{1}X_{3}\\ +0.000683333X_{2}X_{3}-0.011388{X_{1}}^{2}-0.10515{X_{2}}^{2}-0.000793056{X_{3}}^{2} \end{array}$$

Table 2 shows the analysis of variance (ANOVA) for the regression equation. The linear term and quadratic term were highly significant (*p* < 0.01). The model *F*-value of 138.63 implied that the adequate precision measures the signal to noise ratio. There was only a 0.01% chance that a “model *F*-Value” this large could occur due to noise [12]. According to the model *P*-value, X_1_, X_2_, X_3_, X_1_X_2_, X_1_X_3_, X_1_^2^, X_2_^2^ and X_3_^2^ were significant model terms. A non-significant lack of fit *F*-value of 2.25 was also observed, which indicated that the quadratic model was valid for the present study. The coefficient of correlation (*R*^2^) was 0.9944, showing good fitness of the model. Le Man *et al.* [13] and Chauhan and Gupta [14] have emphasized the acceptance of any model with *R*^2^ > 0.75. And the “Pred *R*-Squared” of 0.9407 is in reasonable agreement with the “Adj *R*-Squared” of 0.9872. In addition, the coefficient of variation (CV) was 0.51%, which implied that the model was reproducible.

**Figure 1:**
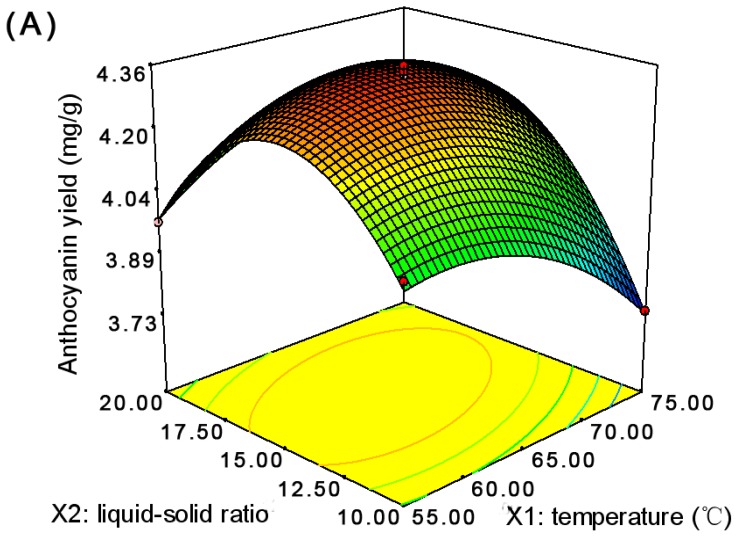

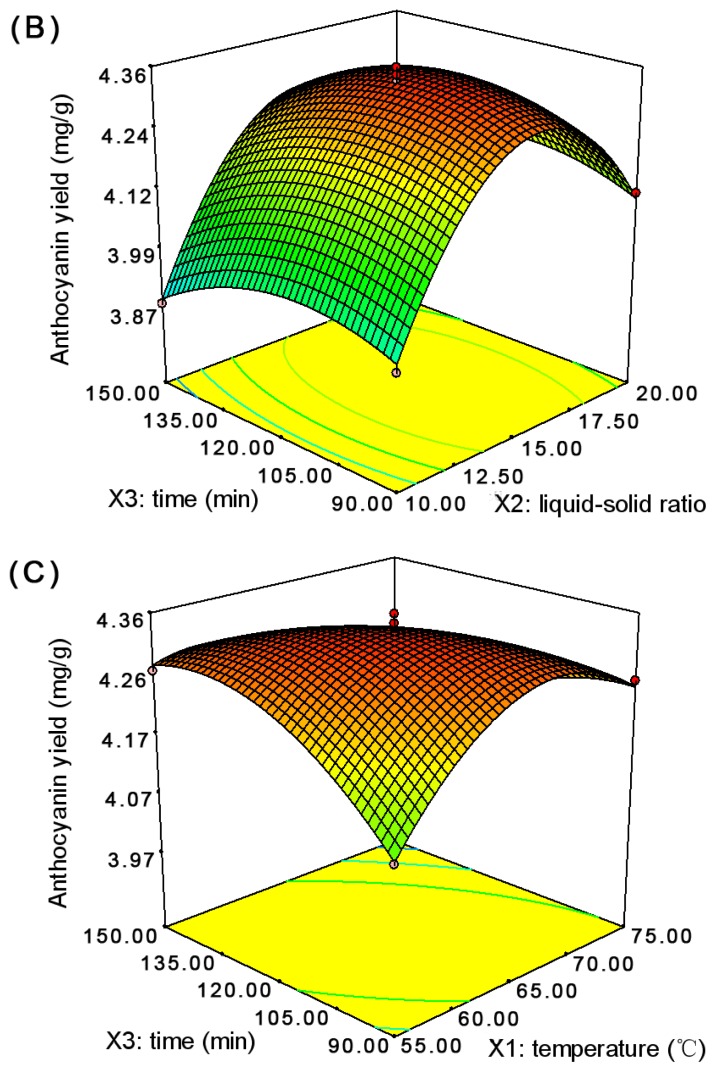
Response surface plots of the anthocyanin yield (Y) of the fruit skin of *Rhodomyrtus tomentosa* (Ait.) Hassk extract as affected by extraction temperature, liquid-solid ratio, and extraction time. (**A**) temperature (X_1_) and liquid-solid ratio (X_2_) at an extraction time of 120 min; (**B**) liquid-solid ratio (X_2_) and time (X_3_) at an extraction temperature of 65 °C; (**C**) temperature (X_1_) and time (X_3_) at a liquid-solid ratio of 15:1.

Surface and contour plots demonstrating the effects of different variables; two variables varied at time while the third is kept constant, on the response function (anthocyanin yield) are shown in Figure 1. The effect of temperature (X_1_) and liquid-solid ratio (X_2_) in the efficiency of extraction are reflected in Figure 1A. When X_1_ was fixed, e.g., at 60 °C, as X_2_ increased, anthocyanin yield (Y) mounted up, and reached the highest value, and then decreased. When X_2_ was fixed, e.g., at 12.5, as X_1_ increased, anthocyanin yield increased gradually, and reached the highest value, and then decreased. The similar relationship between Y and X_2_ as well as time (X_3_) was illustrated in Figure 1B. It indicated that the changes of X_2_ had more significant effects on Y than the X_3_. The combined effect of temperature (X_1_) and time (X_3_) is shown in Figure 1C. It can be seen that, when X_1_ was more than 65 °C, the increase of X_3_ could cause a decrease of Y. Therefore, it could be concluded that the extraction temperature and liquid-solid ratio played more prominent roles on getting high extraction efficiency of anthocyanins during the extraction process.

The optimal values of the selected variables were obtained by solving the regression equation. After calculation by the Design Expert software, the optimal conditions for extracting anthocyanins from the fruit skin of downy rose-myrtle were 64.38 °C, 116.88 min, 15.7:1 liquid-solid ratio, with the corresponding Y = 4.345 mg/g. To confirm the validity of the statistical experimental strategies, a confirmation experiment with a triplicate set was performed at the selected optimum conditions. Verification tests performed gave 4.358 ± 0.045 mg/g, which clearly showed that the model fitted the experimental data and optimized the extraction process.

Our results show that the freeze-dried fruit skins of downy rose-myrtle contain anthocyanin at >4.3 mg/g, indicating that the content in the fresh whole fruit is >153 mg/100 g. Consistent with previous reports [4,15], the present results support the fact that dark berries are a superior source of anthocyanins. The anthocyanin contents in typical berries in Finland were systematically higher than those in this study [15]. However, these results are not comparable as such due to the different quantification methods. The anthocyanin contents in the present study are expressed as the weight of a mixture of relevant anthocyanidin 3-glucosides, whereas the results reported by Koponen and co-workers were expressed for the weight of the aglycones (anthocyanidins). In addition, anthocyanin in the pulp was not determined in the present study. Thus, these anthocyanin content values are only estimates and may be misleading; we are interested in systematically exploring anthocyanin contents and components in different berries in South China in our further studies.

### 2.2. Identification of Anthocyanins in the Extract

**Figure 2:**
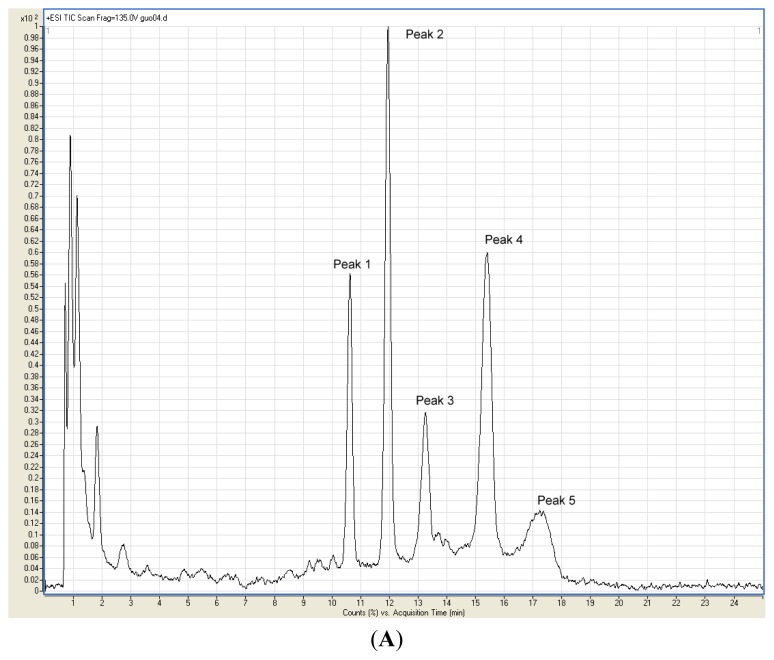

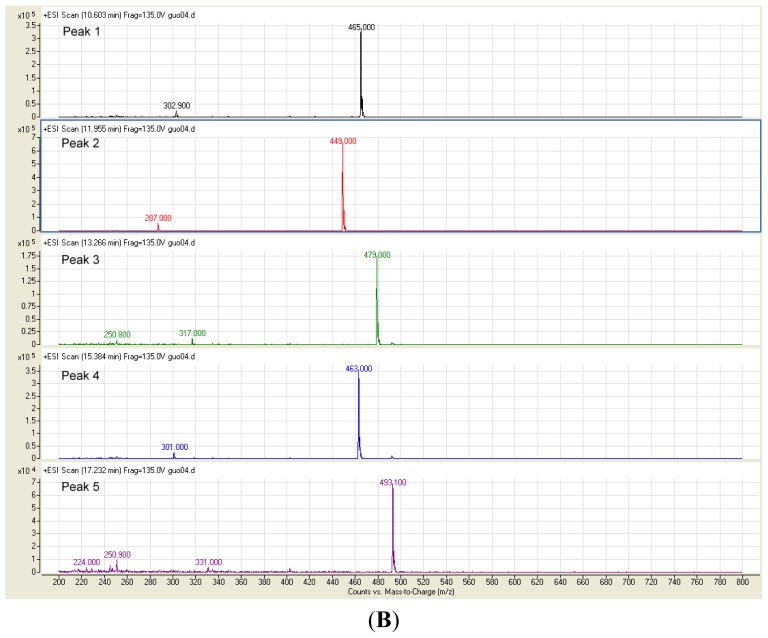
High-performance liquid chromatography-electrospray (HPLC) chromatogram (520 nm) of the anthocyanin-rich extract (**A**) and mass spectrometry (MS) product ions (**B**).

Gradient reversed-phase HPLC with absorbance detection and MS analysis was used to rapidly identify the main anthocyanins in the fruit skin extracts of downy rose-myrtle. The identification was carried out by comparison retention time and mass spectral data with those of standards. Moreover, previous studies of other researchers were taken as reference for us [11,12,16]. There were no significant differences detected in HPLC-MS results among the extracts at different extraction conditions. Figure 2 shows the representative HPLC chromatogram (A) and MS product ions (B) of the anthocyanins extract at λ = 520 nm. The major anthocyanins were tentatively identified as delphinidin-3-glucoside (peak 1), cyanidin-3-glucoside (peak 2), petunidin-3-glucoside (peak 3), peonidin-3-glucoside (peak 4), and malvidin-3-glucoside (peak 5).

Peak 1 (retention time (*t*_R_) = 10.603 min), with M^+^ at *m/z* 465.0, was identified as delphinidin-3-glucoside and a fragment ion at *m/z* 302.9 corresponded to delphinidin. Peak 2 (*t*_R_ = 11.955 min), with M^+^ at *m/z* 449.0, was identified as cyanidin-3-glucoside and a fragment ion at *m/z* 287.0 corresponded to cyanidin. Peak 3 (*t*_R_ = 13.266 min) had a molecular ion *m/z* 479.0 and a fragment ion *m/z* 317.0 indicated that peak 3 was petunidin-3-glucoside. Peak 4 (*t*_R_ = 15.384 min), with M^+^ at *m/z* 463.0, was identified as peonidin-3-glucoside and a fragment ion at *m/z* 301.0 corresponded to peonidin. Peak 5 (*t*_R_ = 17.232 min), with M^+^ at *m/z* 493.1, was identified as malvidin-3-glucoside and a fragment ion at *m/z* 331.0 corresponded to malvidin.

## 3. Experimental Section

### 3.1. Sample Preparation and Chemicals

Fresh mature fruits of downy rose-myrtle variety “Gangren” were obtained in September 2010 from a local market in Shaoguan (Guangdong, China). Skins of fruit samples were separated manually. The skins were dried in a freeze dryer (−40 °C) (ZD-A3, Nanjing, China), pulverized by a disintegrator (FSD-100A, Taizhou, China) and shifted through a 100-mesh sieve. The downy rose-myrtle skin powder (water content <12%) was sealed in a brown bottle and kept at −18 °C until further analysis. Standards of cyanidin-3-glucoside, delphinidin-3-glucoside, petunidin-3-glucoside, peonidin-3-glucoside, and malvidin-3-glucoside were provided by Polyphenol AS (Sandnes, Norway). HPLC grade formic acid and acetonitrile were purchased from Merck (Darmstadt, Germany). All other chemicals were obtained from Guangzhou Chemical Industry (Guangzhou, China).

### 3.2. Extraction of Anthocyanins

Freeze-dried powder samples (1.0 g) were added to a 50-mL Erlenmeyer flask and then mixed with different volumes of 60% ethanol containing 0.1% (v/v) hydrochloric acid (pH~1.0) [17] to give liquid-solid ratios ranging from 10 to 20 (v/w), and put in thermostatic water bath at selected temperatures (55–75 °C) for various durations of time (90–150 min), then centrifuged at 5400 g for 10 min at 25 °C. The supernatant was collected and diluted to 50 mL with the same solvent. All samples were filtered through a 0.45 μm syringe filter (Pall Life Sciences, Ann Arbor, MI, USA). Then, aliquots of those samples were taken to evaluate total anthocyanin content and to identify specific anthocyanin components in the extract.

### 3.3. Total Anthocyanin Content Measurement

The total anthocyanin content was determined according to the spectrophotometric pH-differential method [18]. Briefly, an aliquot (1 mL) of the extract was mixed with 0.025 M potassium chloride buffer (pH 1.0, 4 mL) and 0.4 M sodium acetate buffer (pH 4.5, 4 mL), respectively. The absorbance of the mixture was measured at 510 and 700 nm using a UV-Vis spectrophotometer model UI-trospec 2000 (Amersham Pharmacia Biotech, Dubendorf, Switzerland). Absorbance was calculated as A = [(A_510_ − A_700_) at pH 1.0] − [(A_510_ − A_700_) at pH 4.5] with a molar extinction coefficient of 26,900 for anthocyanin. The total anthocyanin content was calculated as cyanidin-3-glucoside equivalents as Equation (2):

(2) $$\text{Anthocyanin content }(\text{mg}/\text{g})=\frac{A\times MW\times DF\times V\times 10^{3}}{ɛ\times L\times m}$$

where A is absorbance, MW is the molecular weight of cyanidin-3-glucoside (449.2 Da), DF is the dilution factor, V is the final volume (mL), 10^3^ is the factor for conversion from g to mg, ε is the cyanindin-3-glucoside molar absorbance (26,900), L is the cell path length (1 cm), and m is the freeze-dried fruit skin weight (g).

### 3.4. Experimental Design for Optimization

RSM was used to optimize the extraction of anthocyanins from the fruit skin of downy rose-myrtle. A Box-Behnken design was performed using Design-Expert software (Version 7.0, Stat-Ease Inc., Minneapolis, MN, USA, 2008).The independent variables were extraction temperature (55–75 °C), liquid-solid ratio (10–20), and time (90–150 min), while the response variable was the anthocyanin yield. They were termed as X_1_, X_2_, X_3_ and Y, respectively. The experimental results were fitted to the second-order regression (Equation 3):

(3) $$Y=\beta_{0}+\beta_{1}X_{1}+\beta_{2}X_{2}+\beta_{3}X_{3}+\beta_{11}{X_{1}}^{2}+\beta_{22}{X_{2}}^{2}+\beta_{33}{X_{3}}^{2}+\beta_{12}X_{1}X_{2}+\beta_{13}X_{1}X_{3}+\beta_{23}X_{2}X_{3}$$

where Y is the predicted response, β_0_ is the intercept; β_1_, β_2_, β_3_ are linear coefficients; β_11_, β_22_, β_33_ are squared coefficients; β_12_, β_13_, β_23_ are interaction coefficients. The goodness-of-fit of the regression model and the significance of parameter estimates were determined by the analysis of variance (ANOVA).

### 3.5. Identification of Anthocyanins in Fruit Skin Extract by HPLC-ESI-MS

An Agilent 1200 series high-performance liquid chromatography (Agilent Technologies, Palo Alto, CA, USA) coupled to an Agilent 6410 triple quadrupole mass spectrometer and an Agilent Zorbax SB-C18 column (1.8-μm; 2.1 mm × 50 mm) was used to identify anthocyanins in the fruit skin extract [16,19].

Chromatographic separation was performed using a mixture of two eluents: solvent A, 5% formic acid in water (V/V); and solvent B, acetonitrile. The gradient elution program was used as follows: 0 to 2 min, 6% B; 2 to 8 min, 6% to 12% B; 8 to 20 min, 12% to 20% B; 20 to 28 min, 20% B. Column temperature was 25 °C, flow rate was 0.5 mL/min, and injection volume was 2 μL.

For the identification of anthocyanins, electrospray ionization (ESI) was operated in the positive ion mode in the mass range 200 to 800 (*m/z*) and under the following conditions: capillary voltage, 4500 V; nebulizer pressure, 40 psi; drying gas temperature at 350 °C was used at a flow rate of 8.0 L/min.

### 3.6. Statistical Analysis

All the experiments were carried out in triplicate, and the results are expressed as means ± SD (standard deviation). Statistical analyses were performed using the SPSS 14.0 package (SPSS Inc., Chicago, IL, USA, 2005). A value of *p* < 0.05 was considered statistically significant.

## 4. Conclusions

The experimental design approach, RSM, was successfully applied in the optimization of the extraction of anthocyanins from the fruit skin of *Rhodomyrtus tomentosa* (Ait.) Hassk var. Gangren. Using 60% ethanol containing 0.1% (v/v) hydrochloric acid as solvent, the predicted process variables for best combination of response function were temperature 64.38 °C, time 116.88 min, and liquid-solid ratio 15.7:1, which may be useful for industrial extraction of anthocyanin from the berry of downy rose-myrtle. In addition, five anthocyanins were tentatively identified in the fruit skin extract, namely cyanidin-3-glucoside, delphinidin-3-glucoside, peonidin-3-glucoside, petunidin-3-glucoside and malvidin-3-glucoside.

## Figures and Tables

**Table 1:** Box-Behnken design arrangement and response.

Run	Temperature (°C)	Liquid-Solid Ratio (v/w)	Time (min)	Anthocyanin Yield a (mg/g)	

				Observed	Predicted
1	65	20	150	4.088	4.071
2	65	15	120	4.358	4.337
3	55	10	120	4.048	4.027
4	75	10	120	3.737	3.730
5	55	15	150	4.268	4.278
6	75	15	150	3.983	3.979
7	55	20	120	3.968	3.975
8	65	10	150	3.877	3.888
9	65	15	120	4.343	4.337
10	65	15	120	4.313	4.337
11	65	10	90	3.938	3.955
12	65	15	120	4.328	4.337
13	55	15	90	4.103	4.107
14	75	20	120	4.088	4.109
15	65	20	90	4.108	4.097
16	75	15	90	4.253	4.243
17	65	15	120	4.343	4.337

a Data are presented as mean of triplicate analyses.

**Table 2:** Analysis of Variance (ANOVA) for the regression equation.

Source	Sum of Squares	df	Mean Square	*F*-Value	*p*-Value
Model	0.5600	9	0.0620	138.63	<0.0001
X_1_	0.0130	1	0.0130	29.55	0.0010
X_2_	0.0530	1	0.0530	118.22	<0.0001
X_3_	0.0043	1	0.0043	9.62	0.0173
X_1_X_2_	0.0460	1	0.0460	103.32	<0.0001
X_1_X_3_	0.0470	1	0.0470	105.24	<0.0001
X_2_X_3_	0.0004	1	0.0004	0.93	0.3658
X_1_^2^	0.0550	1	0.0550	121.47	<0.0001
X_2_^2^	0.2900	1	0.2900	647.30	<0.0001
X_3_^2^	0.0210	1	0.0210	47.72	0.0002
Residual	0.0031	7	0.0004		
Lack of Fit	0.0020	3	0.0006	2.25	0.2245
Pure Error	0.0012	4		0.0002	
Cor Total	0.5600	16			

*R*^2^ = 0.9944; CV = 0.51%; CV, coefficient of variation; df = degree of freedom; X_1_: temperature; X_2_: liquid-solid ratio; X_3_: time.

## References

[1] Nabae K., Hayashi S.M., Kawabe M., Ichihara T., Hagiwara A., Tamano S., Tsushima Y., Uchida K., Koda T., Nakamura M. (2008). A 90-day oral toxicity study of purple corn color, a natural food colorant, in F344 rats. Food Chem. Toxicol.

[2] He J., Giusti M.M. (2010). Anthocyanins: Natural colorants with health-promoting properties. Annu. Rev. Food Sci. Technol.

[3] Felgines C., Talavera S., Texier O., Besson C., Fogliano V., Lamaison J.L., la Fauci L., Galvano G., Remesy C., Galvano F. (2006). Absorption and metabolism of red orange juice anthocyanins in rats. Br. J. Nutr.

[4] Wu X., Beecher G.R., Holden J.M., Haytowitz D.B., Gebhardt S.E., Prior R.L. (2006). Concentrations of anthocyanins in common foods in the United States and estimation of normal consumption. J. Agric. Food Chem.

[5] Ghosh D., Konishi T. (2007). Anthocyanins and anthocyanin-rich extracts: Role in diabetes and eye function. Asia Pac. J. Clin. Nutr.

[6] Saising J., Ongsakul M., Voravuthikunchai S.P. (2011). *Rhodomyrtus tomentosa* (Aiton) Hassk. ethanol extract and rhodomyrtone: A potential strategy for the treatment of biofilm-forming staphylococci. J. Med. Microbiol.

[7] Bagchi D., Roy S., Patel V., He G., Khanna S., Ojha N., Phillips C., Ghosh S., Bagchi M., Sen C.K. (2006). Safety and whole-body antioxidant potential of a novel anthocyanin-rich formulation of edible berries. Mol. Cell. Biochem.

[8] Bechtold T., Mahmud-Ali A., Mussak R. (2007). Anthocyanin dyes extracted from grape pomace for the purpose of textile dyeing. J. Sci. Food Agric.

[9] Mundra P., Desai K., Lele S.S. (2007). Application of response surface methodology to cell immobilization for the production of palatinose. Bioresour. Technol.

[10] Ku C.S., Mun S.P. (2008). Optimization of the extraction of anthocyanin from Bokbunja (*Rubus coreanus* Miq.) marc produced during traditional wine processing and characterization of the extracts. Bioresour. Technol.

[11] Zou T.B., Wang M., Gan R.Y., Ling W.H. (2011). Optimization of ultrasound-assisted extraction of anthocyanins from mulberry, using response surface methodology. Int. J. Mol. Sci.

[12] Yang Z., Zhai W. (2010). Optimization of microwave-assisted extraction of anthocyanins from purple corn (*Zea mays* L.) cob and identification with HPLC-MS. Innov. Food Sci. Emerg.

[13] Le Man H., Behera S., Park H. (2010). Optimization of operational parameters for ethanol production from Korean food waste leachate. Int. J. Environ. Sci. Technol.

[14] Chauhan B., Gupta R. (2004). Application of statistical experimental design for optimization of alkaline protease production from *Bacillus* sp. RGR-14. Process. Biochem.

[15] Koponen J.M., Happonen A.M., Mattila P.H., Torronen A.R. (2007). Contents of anthocyanins and ellagitannins in selected foods consumed in Finland. J. Agric. Food Chem.

[16] Nicoue E.E., Savard S., Belkacemi K. (2007). Anthocyanins in wild blueberries of Quebec: Extraction and identification. J. Agric. Food Chem.

[17] Guo H., Ling W., Wang Q., Liu C., Hu Y., Xia M., Feng X., Xia X. (2007). Effect of anthocyanin-rich extract from black rice *(Oryza sativa* L. *indica*) on hyperlipidemia and insulin resistance in fructose-fed rats. Plant Foods Hum. Nutr.

[18] Lee J., Durst R.W., Wrolstad R.E. (2005). Determination of total monomeric anthocyanin pigment content of fruit juices, beverages, natural colorants, and wines by the pH differential method: Collaborative study. J. AOAC Int.

[19] Zou T., Wang D., Guo H., Zhu Y., Luo X., Liu F., Ling W. (2012). Optimization of microwave-assisted extraction of anthocyanins from mulberry and identification of anthocyanins in extract using HPLC-ESI-MS. J. Food Sci.

